# Follow-up blood cultures in *Pseudomonas aeruginosa* bacteremia: A potential target for diagnostic stewardship

**DOI:** 10.1017/ash.2021.184

**Published:** 2021-08-27

**Authors:** Alexis L. Green, Yuanyuan Liang, Lyndsay M. O’Hara, Lisa Pineles, Scott Sorongon, Anthony D. Harris, Jonathan D. Baghdadi

**Affiliations:** 1 University of Maryland School of Medicine, Baltimore, Maryland; 2 Department of Epidemiology and Public Health, University of Maryland School of Medicine, Baltimore, Maryland

**Keywords:** Pseudomonas aeruginosa, follow-up blood cultures, diagnostic stewardship

## Abstract

**Objectives:**

Evidence supporting collection of follow-up blood cultures for Gram-negative bacteremia is mixed. We sought to understand why providers order follow-up blood cultures when managing *P. aeruginosa* bacteremia and whether follow-up blood cultures in this context are associated with short- and long-term survival.

**Methods:**

We conducted a retrospective cohort study of adult inpatients with *P. aeruginosa* bacteremia at the University of Maryland Medical Center in 2015–2020. Kaplan-Meier survival curves and Cox regression with time-varying covariates were used to evaluate the association between follow-up blood cultures and time to mortality within 30 days of first positive blood culture. Provider justifications for follow-up blood cultures were identified through chart review.

**Results:**

Of 159 eligible patients, 127 (80%) had follow-up blood cultures, including 9 (7%) that were positive for *P. aeruginosa* and 10 (8%) that were positive for other organisms. Follow-up blood cultures were typically collected “to ensure clearance” or “to guide antibiotic therapy.” Overall, 30-day mortality was 25.2%. After risk adjustment for patient characteristics, follow-up blood cultures were associated with a nonsignificant reduction in mortality risk (hazard ratio, 0.43; 95% confidence interval, 1.08; *P* = .071). In exploratory analyses, the potential mortality reduction from follow-up blood cultures was driven by their use in patients with Pitt bacteremia scores >0.

**Conclusions:**

Follow-up blood cultures are commonly collected for *P. aeruginosa* bacteremia but infrequently identify persistent bacteremia. Targeted use of follow-up blood cultures based on severity of illness may reduce unnecessary culturing.

Gram-negative bacteremia is associated with high mortality.^1^ Although follow-up blood cultures are recommended in the management of bacteremia due to Gram-positive organisms such as *Staphylococcus aureus*, our understanding of their role in Gram-negative bacteremia is evolving.^2^ Early studies of Gram-negative bacteremia caused predominantly by *Escherichia coli* suggested that follow-up blood cultures are rarely positive and do not correlate well with clinical outcomes.^3,4^ Subsequent studies including larger samples of patients with bacteremia due to other Gram-negative organisms have demonstrated higher rates of persistent bacteremia and a potential mortality benefit when follow-up blood cultures are collected.^5,6^ No studies have focused exclusively on *Pseudomonas aeruginosa.*

Our institution has recently begun an initiative related to diagnostic stewardship of blood cultures, including implementation of an algorithm to guide when culturing is appropriate for fever in the intensive care unit. Although infections due to *P. aeruginosa* are associated with higher mortality than infections due to other Gram-negative organisms,^7^ *P. aeruginosa* may be less likely than other Gram-negative organisms, such as *Serratia* spp or *Stenotrophomonas maltophilia*, to be associated with persistent bacteremia.^6^ With adequate source control, follow-up blood cultures may be unnecessary.^8^ To support diagnostic stewardship of blood cultures at our institution, we evaluated the utility of follow-up blood cultures in cases of *P. aeruginosa* bacteremia by determining their positivity rate and association with 30-day mortality. In secondary analyses, we explored which patient groups were most likely to benefit from follow-up blood cultures.

## Methods

### Study population

The cohort included all adult patients admitted to the University of Maryland Medical Center between November 1, 2015, and January 1, 2020, with a blood culture positive for *P. aeruginosa*. Patients who died or were discharged within 24 hours of the initial blood culture were excluded. For patients who had >1 hospital stay in which they had a positive *P. aeruginosa* blood culture, only the first hospital stay was included. This study was deemed exempt from review by the University of Maryland Baltimore Institutional Review Board.

### Data collection

Data were extracted from the electronic medical records of each patient and supplemented with chart review. All data elements extracted electronically were validated by chart review in a random sample of 20 charts. The variables we investigated included demographic information (age, sex, race, and BMI), clinical characteristics (source of bacteremia, presence of a Foley catheter or central line at the time of the first positive blood culture), and components of the Pitt bacteremia score (temperature, blood pressure, cardiac arrest, mechanical ventilation, and mental status).^9,10^ The primary outcome was time in days from the first positive blood culture to all-cause mortality for up to 30 days of follow-up. A 30-day follow-up period was chosen to capture clinical outcomes during the acute phase of illness which might occur after hospital discharge. Mortality events were identified through review of connected electronic health records available on the Care Everywhere Network provided by Epic and review of records from CRISP, the designated health information exchange for the state of Maryland.^11^ Individuals who did not have documented follow-up at least 30 days after the index positive blood culture were censored at the time of last documented living interaction with healthcare. Individuals who had documented living interaction 30 days after the index positive blood culture were censored at 30 days. A sensitivity analysis was performed using time from the first positive blood culture to mortality within a 90-day follow-up period as the outcome.

Source of bacteremia was determined by review of progress notes from the infectious disease consultant or primary team. Charts were independently reviewed by 2 members of the study team (A.G. and J.B.) in sets of 10, with a third team member available for adjudication (A.H.). Once agreement between the 2 reviewers reached >90%, remaining charts were reviewed by a single individual (A.G.). Documented justification for follow-up blood culture was collected from the progress note of the infectious disease consultant or primary team.

### Definitions

Blood cultures were categorized as follow-up if collected >24 hours and ≤7 days after the index positive, in accordance with the literature.^3^ In time-to-event analysis, the exposure of interest was a time-varying binary variable representing the presence of follow-up blood culture at a given time after the index positive blood culture (time 0). Physician justification for follow-up blood culture was defined by documentation of a reason for follow-up blood cultures on the day before, day of, or day after collection. Baseline health was represented using the sum of conditions present from the Elixhauser comorbidity index as defined using *International Classification of Disease, Tenth Revision* (ICD-10) codes.^12^ Hospital-onset bacteremia was defined by the index positive blood culture occurring after hospital day 3. The Pitt bacteremia score was calculated using the most abnormal values for each contributing element from the calendar day of the index positive blood culture. In multivariable analyses, Pitt bacteremia scores were categorized as low (a score of 0), moderate (scores 1–4), and high (scores ≥5) based on thresholds from the literature used to indicate severe infection.^13^ Antimicrobial resistance, selection of antibiotic coverage, and use of empiric therapy were captured in a single variable representing the number of days from collection of the index positive blood culture to administration of effective antibiotic therapy. Antibiotics administered >24 hours before collection of the index positive blood culture were excluded. In multivariable analyses, days to effective antibiotic therapy was categorized into 4 levels based on whether effective antibiotics were started concurrently with the index blood culture (from 24 hours before until 3 hours after), within 1 day (3–24 hours after the index blood culture), within 2 days (24–48 hours after the index blood culture), or after a delay of >48 hours.

### Statistical analysis

Patient demographics and clinical characteristics were described using means and standard deviations or frequencies and proportions, as appropriate. Comparisons between patients who underwent follow-up blood culture and those who did not were performed using the Student *t* test or Wilcoxon rank-sum test for continuous variables and Pearson χ^2^ test or Fisher exact test for categorical variables. Logistic regression was used to evaluate associations between predictors and binary outcomes, such as positivity of follow-up blood culture.

The cumulative incidence of mortality for each time-varying follow-up blood culture group was presented using Kaplan-Meier survival curves. The association between follow-up blood culture collection and time to mortality within the period of follow-up (30 days or 90 days) was modeled using Cox proportional hazards regression while adjusting for confounding variables. To explore the potential impact of follow-up blood cultures in patients with different levels of severity of illness, heterogeneity of treatment effect analyses were performed by including an interaction term between follow-up blood cultures and Pitt bacteremia scores in the Cox model.

In all time-to-event analyses, the presence of follow-up blood culture was defined as a time-varying binary variable to account for immortal time bias.^14^ We estimated 95% confidence intervals (CIs) for hazard ratios (HRs) using robust standard errors. Covariates included in the Cox model for risk adjustment included age, baseline health in terms of number of conditions from the Elixhauser comorbidity index, Pitt bacteremia score, presence of immunosuppression, hospital-onset of index bacteremia, and days from index positive blood culture collection to administration of effective antibiotics. Statistical analyses were performed with SAS version 9.4 software (SAS Institute, Cary, NC) and Stata/SE version 16 software (StataCorp, College Station, TX).

### Patient consent statement

This study was determined to be exempt from requirements for patient consent by the Institutional Review Board of the University of Maryland School of Medicine. A waiver of HIPAA authorization for release of the private health information was approved.

## Results

### Patient characteristics and follow-up blood cultures

**Fig. 1.**
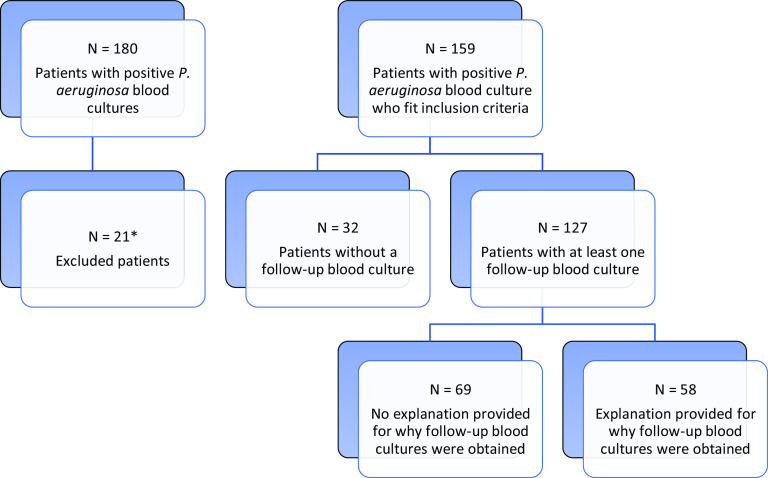
Flow chart depicting patient selection (excluding explanation for obtaining FUBC). *Patients were excluded if they died or were discharged within 24 hours of first positive blood culture, if they had a previous admission with a positive blood culture, or if the positive blood culture date preceded the admission date.

In total, 180 patients with a positive *P. aeruginosa* blood culture were identified, including 159 unique patients who met the inclusion criteria and 127 patients (80% of 159) with at least 1 follow-up blood culture (see Fig. 1 for patient flow diagram). Patients who had follow-up blood cultures were similar to those who did not (Table 1). All 23 patients whose bacteremia was attributed to an indwelling vascular catheter had follow-up blood cultures obtained. Among them, 74.2% of patients received effective antibiotics within 24 hours of the index positive blood culture (median time to effective antibiotics, 4.7 hours; IQR, 0.7–25.0). In addition, >90% of patients were seen by an infectious disease consultant within 48 hours of the index positive blood culture, regardless of whether follow-up blood cultures were obtained.

**Table 1.** Description of Patients With *Pseudomonas aeruginosa* Bacteremia (N=159)

Variable	All Patients (n=159) No. (%)	Follow-Up Blood Culture (n=127) No. (%)	No Follow-Up Blood Culture (n=32) No. (%)	*P* Value
Age, mean y (SD)	56.6 (16.3	56.9 (16.5)	59.3 (15.3)	.21
Sex, male	94 (59.1)	76 (59.8)	18 (56.3)	.71
**Race**				
Black or African American	71 (44.7)	58 (45.7)	13 (40.6)	.22
White	77 (48.4)	63 (49.6)	14 (43.8)	
Hispanic	3 (1.9)	2 (1.6)	1 (3.1)	
≥2 races	1 (0.6)	1 (0.8)	0	
Declined to answer or unknown	7 (4.4)	3 (2.4)	4 (12.5)	
BMI (mean, SD)	28.1, 7.2	28.3, 7.0	27.1, 8.1	.41
Elixhauser score (median, IQR)	5 (4–7)	5 (4–7)	4.5 (3–7)	.48^a^
Immunosuppressant agents	90 (56.6)	69 (54.3)	21 (65.6)	.25
Hemodialysis dependence	31 (19.5)	23 (18.1)	8 (25.0)	.38
ICU care	73 (45.1)	60 (46.2)	13 (40.6)	.57
Foley catheter	58 (36.5)	46 (36.2)	12 (37.5)	.89
Mechanical ventilation	55 (34.8)	43 (34.1)	12 (37.5)	.72
Central line	94 (59.1)	77 (60.6)	17 (53.1)	.44
PITT bacteremia score, median (IQR)	2 (1–4)	2 (0–4)	3 (0.5–5.5)	.16^a^
Hours from blood culture collection to effective antibiotics, median (IQR)	4.7 (0.7–25.0)	4.1 (0.7–24.0)	6.4 (0.8–38.3)	.35^a^
ID consult <24 h after bacteremia	153 (95.6)	121 (95.3)	31 (96.9)	.69
**Source of bacteremia**				.001
Line related	23 (14.5)	23 (18.1)	0 (0.0)	
Urinary system infection	17 (10.7)	13 (10.2)	4 (12.5)	
Nonurinary intra-abdominal infection	27 (17.0)	14 (11.0)	13 (40.6)	
Pneumonia	22 (13.8)	17 (13.4)	5 (15.6)	
Multiple sources	6 (3.8)	6 (4.7)	0 (0.0)	
Other	24 (15.1)	22 (17.3)	2 (6.3)	
Unknown	40 (25.2)	32 (25.2)	8 (25.0)	

Note. SD, standard deviation; IQR, interquartile range.

a Mann-Whitney *U* test.

The median number of follow-up blood cultures was 1 (maximum, 5). The median time to first follow-up blood culture was 52.0 hours (IQR, 32.5–100.9). Follow-up blood cultures were positive in 18 patients (14.2% of 127), including 9 cases of persistent Pseudomonal bacteremia (7.1%). Follow-up blood cultures were positive for *Candida* spp in 4 cases, *Klebsiella pneumoniae* in 3 cases, and other organisms in 4 cases. In unadjusted analyses, presence of a central line (OR, 0.78; 95% CI, 0.29–2.1; *P* = .20), dependence on hemodialysis (OR, 1.35; 95% CI, 0.40–4.6; *P* = .63), Pitt bacteremia score (OR, 0.89; 95% CI, 0.73–1.10; *P* = .29), and days from index positive blood culture collection to administration of effective antibiotics (OR, 1.22; 95% CI, 0.83–1.80 per 1-day increase; *P* = .31) were not significantly associated with isolation of a pathogen from follow-up blood culture.

Among the 94 patients with a central line present before onset of bacteremia, 74 (79%) had their line removed during the 30-day follow-up period, and 32 (34%) had their line removed before completing inpatient antibiotics. The median time to line removal was 63 hours (IQR, 23–192). Among 77 patients with follow-up blood cultures, 30 (39%) had their central lines removed before follow-up blood cultures were obtained. Of 30 patients with line removal prior to follow-up blood culture, 3 (10%) were still bacteremic at time of follow-up, compared with 7 (15%) of 47 patients whose line remained in place (*P* = .533, χ^2^ test).

### Reasons for obtaining follow-up blood cultures

**Fig. 2.**
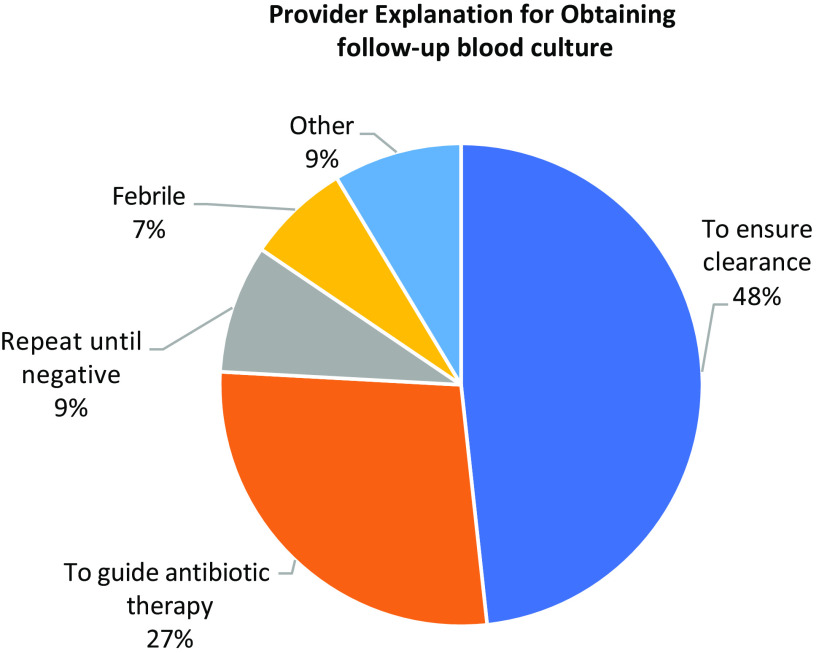
Provider explanation for obtaining follow-up blood culture (N = 58).

A justification for follow-up blood cultures was documented in the charts of 58 (45.7%) of 127 eligible patients (Fig. 2). The most common justifications for follow-up blood cultures were to ensure clearance (28 cases), to guide antibiotic therapy (16 cases), “repeat until negative” (5 cases), and in response to fever (4 cases). Justification for follow-up blood culture was associated with a positive overall impression of the patient’s condition in 20 cases, a negative impression in 31 cases, and no assessment of the patient’s condition in 7 cases. Follow-up blood cultures to guide antibiotic therapy (10 cases for negative impression, 3 for positive, 3 with no impression) and fever (3 for negative impression, 0 for positive, 1 with no impression) were recommended more frequently in the setting of provider concern. Follow-up blood cultures to ensure clearance or to be repeated until negative were used equally regardless of providers’ overall impression of the patient’s condition.

### Mortality among patients with and without follow-up blood cultures

Unfortunately, 7 patients provided incomplete follow-up data and were censored before 30 days of follow-up (see Supplementary Appendix online for the characteristics of these patients). Also, 40 patients (25.2%) died within 30 days of their index positive blood culture, including 24 (18.9%) of 127 patients who had follow-up blood cultures and 16 (50%) of 32 patients without follow-up blood cultures. Mortality rates within the 30-day follow-up period were similar between patients whose follow-up blood cultures were negative and positive (24.1% vs 33.3%; *P* = .40, χ^2^ testing).

**Fig. 3.**
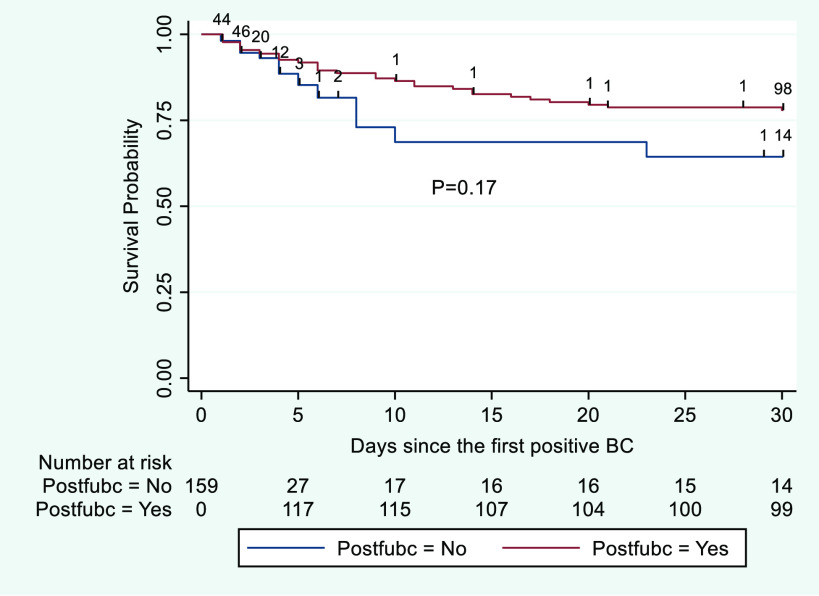
Kaplan-Meier survival curves for patients with and without follow-up blood cultures within 30 days of follow-up. Note. BC, blood cultures. Postfubc indicates status-post follow-up blood cultures.

Among those who died, median times from index positive blood culture to death in the overall sample, patients with follow-up blood culture, and patients without follow-up blood culture were 6 days (IQR, 3–12), 9.5 days (IQR, 5.5–15), and 3.5 (IQR, 2–7), respectively. Kaplan-Meier survival curves for the 30-day follow-up period are displayed in Figure 3. In unadjusted Cox regression, collection of follow-up blood cultures was not significantly associated with risk of mortality (HR, 0.59; 95% CI, 0.26–1.32; *P* = .20). After adjusting for age, baseline health in terms of number of conditions from the Elixhauser comorbidity index, Pitt bacteremia score, presence of immunosuppression, hospital-onset of index bacteremia, and days from index positive blood culture collection to administration of effective antibiotics, follow-up blood cultures remained nonsignificantly associated with risk of mortality (HR, 0.43; 95% CI, 0.17–1.08; *P* = .07) (see Supplementary Appendix online for full model).

### Follow-up blood cultures for patients with low, medium, and high Pitt bacteremia scores

**Fig. 4.**
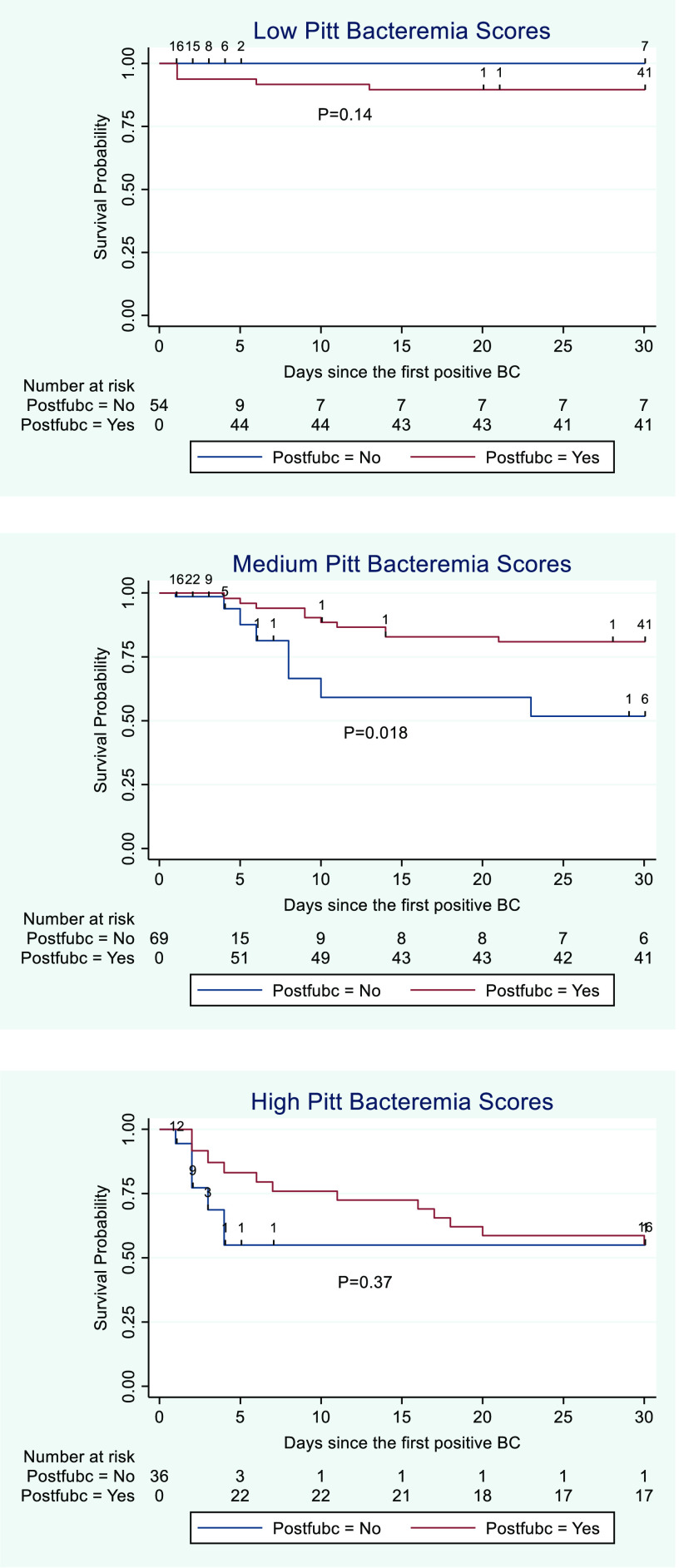
Kaplan-Meier survival curves for patients with and without follow-up blood cultures, stratified by low, medium, and high Pitt bacteremia scores. Postfubc indicates status-post follow-up blood cultures. Low Pitt bacteremia score was defined by a score of 0. Medium scores were 1–4. High scores were ≥5.

In risk-adjusted Cox regression, medium (HR, 4.81; 95% CI, 1.38–16.8; *P* = .014) and high (HR, 15.6; 95% CI, 3.77–64.4; *P* < .001) Pitt bacteremia scores were independently associated with increasing risk of mortality relative to a Pitt bacteremia score of 0 (see Supplementary Appendix online). The Kaplan-Meier survival curves for patients with low, medium, and high Pitt bacteremia scores are displayed in Figure 4. When comparing survival curves by log-rank test without adjustment for confounding variables, follow-up blood cultures were associated with increased survival among patients with medium Pitt bacteremia scores (*P* = .018) (Fig. 4).

Follow-up blood cultures were positive in 10 (21.7%) of 46 patients with Pitt bacteremia scores of zero, 6 (11.1%) of 54 patients with Pitt bacteremia scores 1–4, and 2 (7.4%) of 27 patients with Pitt bacteremia scores >4. Sources of infection for patients with low, medium, and high Pitt scores are reported in the Supplementary Material (online). Follow-up blood cultures were positive for 2 of the 3 patients with low Pitt scores who died within 30 days of their index blood culture, 3 of 10 patients with medium Pitt scores who died within 30 days, and 1 of 11 with high Pitt scores who died within 30 days.

### Sensitivity analysis using 90-day period of follow-up

**Fig. 5.**
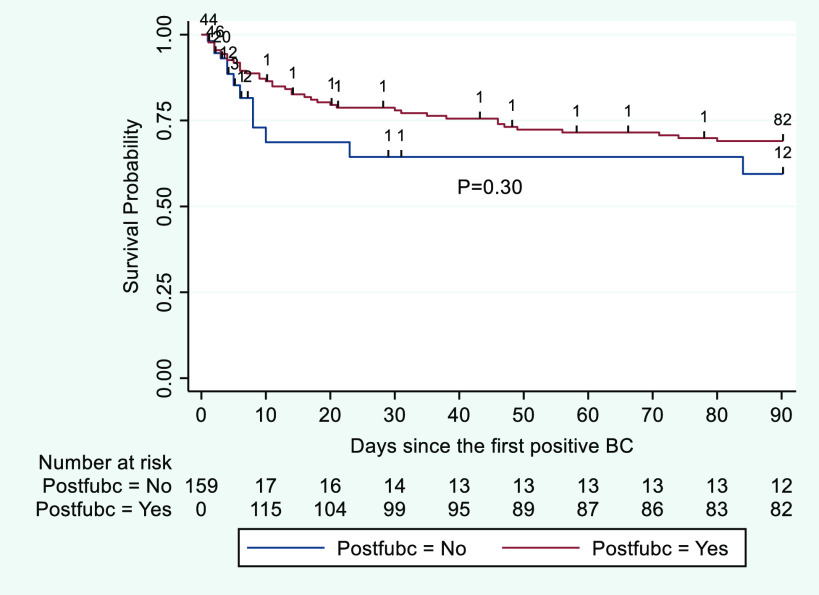
Kaplan-Meier survival curves for patients with and without follow-up blood cultures in 90 days of follow-up. Note. BC, blood cultures. Postfubc indicates status-post follow-up blood cultures.

Kaplan-Meier curves for an extended period of follow-up are displayed in Figure 5. These curves were not significantly different by log-rank test (*P* = .30). Follow-up blood cultures were not significantly associated with risk of mortality in unadjusted Cox regression (HR, 0.70; 95% CI, 0.34–1.44; *P* = .33) or adjusted Cox regression (HR, 0.56; 95% CI, 0.25–1.27; *P* = .17).

## Discussion

Unnecessary blood cultures can waste resources, complicate patient follow-up, and, when positive for a contaminant, adversely affect hospital quality metrics. In this cohort study of 5 years of clinical data from a large medical center, follow-up blood cultures for *P. aeruginosa* bacteremia were routine, but persistent bacteremia was infrequent. Follow-up blood cultures were not significantly associated with improved mortality, though exploratory analyses suggested a potential benefit when they were drawn for patients with elevated Pitt bacteremia scores.

Strategies to improve use of follow-up blood cultures may focus on the underlying infectious syndrome, the pathogen, the adequacy of treatment, or the patient’s condition. In this study, we focused on a pathogen that has been identified as an independent risk factor for breakthrough bacteremia and for which the evidence is limited.^17^ When persistent bacteremia was found to be infrequent, we performed exploratory analyses to identify patients who might be most likely to benefit from follow-up blood cultures, and we investigated potential differences in impact associated with severity of illness. We also explored the reasons why follow-up blood cultures were being obtained and found that they were frequently reflexive, (“repeat until negative”) rather than ordered in response to the patient’s condition.

Repeat blood cultures have been identified as a potentially unnecessary testing practice representing an opportunity for diagnostic stewardship.^15^ At our medical center, follow-up blood cultures are collected in 4 of 5 cases of *P. aeruginosa* bacteremia. Although others have reported similar rates of repeated cultures,^6^ medical centers vary, suggesting that approaches may be cultural rather than evidence based.^4,16^ We suspect that follow-up blood cultures are not necessary in all cases of *P. aeruginosa* bacteremia but have been unable to reach a local consensus on how best to de-implement this process. Thus, when consulted, infectious disease physicians continue to encourage collection of follow-up blood cultures in many cases. Despite recent adoption of protocols to reduce unnecessary “pan-culturing” in the intensive care unit, these protocols explicitly permit repeat cultures to ensure clearance.

Surprisingly, follow-up blood cultures were less frequently positive for patients with elevated Pitt scores. This finding may reflect an increased frequency of line-related bacteremia among the patients with low Pitt scores (see Supplementary Appendix online). However, despite a lower positivity rate, follow-up blood cultures were associated with a trend toward improved survival among patients with Pitt scores >0. Although subgroups with medium and high Pitt scores were small and clinical courses were heterogeneous, we suspect that follow-up blood cultures may support effective clinical management of patients who are severely ill with *P. aeruginosa* bacteremia. Positivity rate is often used as a proxy for clinical utility, but blood cultures may provide clinical utility when negative, particularly among the critically ill. Further investigation, including ideally a larger sample of severely ill patients with pseudomonal bacteremia, is needed before the optimal context for use of follow-up blood cultures can be identified.

Notably, follow-up blood cultures in our cohort were as frequently positive for *P. aeruginosa* as they were for unrelated but nonetheless clinically relevant pathogens. This finding may reflect the high rate of early effective antibiotic therapy or may indicate that *P. aeruginosa* bacteremia is a marker among hospitalized patients for underlying predisposition toward severe infections. Given that mortality was high (25.2%) regardless of whether persistent bacteremia was present, the poor prognosis associated with *P. aeruginosa* bacteremia may be attributable to host factors in addition to acute illness.

This study has several limitations. First, we analyzed observational data from a single medical center, and our findings therefore may not be generalizable to other clinical settings. However, in the context of other single-center studies in the literature, these results are meaningful. Second, though we adjusted for severity of illness using the Pitt bacteremia score and baseline health using the Elixhauser comorbidity index, our results are likely still susceptible to residual confounding related to severity of illness. Third, most follow-up blood cultures in our cohort were drawn within 72 hours of the index positive blood culture. Thus, our results may not be generalizable to clinical protocols that emphasize collection of follow-up blood cultures later in the course of illness or at the conclusion of therapy. Finally, the retrospective design of this study did not permit an evaluation of causal pathways. Rather, we were limited to reporting associations only.

In this retrospective observational study, follow-up blood cultures were not associated with clear or consistent benefit in either short- or long-term outcomes. Follow-up blood cultures were frequently drawn reflexively “to ensure clearance.” Diagnostic stewardship initiatives can potentially reduce unnecessary culturing by targeting follow-up blood cultures based on severity of illness.

## Supplementary material

For supplementary material accompanying this paper visit https://doi.org/10.1017/ash.2021.184. click here to view supplementary material
