# Supplementary material for: Follow-up blood cultures in Pseudomonas aeruginosa bacteremia: A potential target for diagnostic stewardship
Source: Antimicrob Steward Healthc Epidemiol. 2021 Aug 27;1(1):e23. doi: 10.1017/ash.2021.184 (PMC9495539; doi:10.1017/ash.2021.184)
Supplement: Supplementary file 1 [file S2732494X21001844sup001.docx]

**Supplementary Table 1.** Characteristics of Patients Who Were Censored Before 30 Days of Follow-up

| Length of Follow-Up, Days | Age, y / Sex | Pitt  Bacteremia  Score | Elixhauser Comorbidity Index |
| --- | --- | --- | --- |
| 5 | 82 / F | 0 | 1 |
| 10 | 70 / M | 1 | 1 |
| 14 | 59 / F | 1 | 0 |
| 20 | 64 / F | 0 | 1 |
| 21 | 75 / F | 0 | 1 |
| 28 | 37 / M | 4 | 0 |
| 29 | 61 / F | 4 | 1 |

**Supplementary Table 2.** Multivariable Cox Regression Evaluating 30-day Mortality Risk Associated with Follow-up Blood Culture Collection and Other Variables

| Variable | Hazard Ratio (95% CI) | *P*  Value |
| --- | --- | --- |
| Follow-up blood culture obtained | 0.43 (0.17–1.08) | .071 |
| Age | 1.01 (0.98–1.04) | .38 |
| **Pitt score** |  |  |
| 0 | [reference] |  |
| 1–4 | 4.81 (1.38–16.8) | .014 |
| >4 | 15.6 (3.77–64.4) | <.001 |
| Elixhauser | 1.02 (0.88–1.17) | .83 |
| Immunosuppression | 1.14 (0.62–2.08) | .68 |
| Index blood culture collected >3 days after admission | 0.46 (0.20–1.06) | .068 |
| **Time to effective antibiotics** |  |  |
| Within 3 h | [reference] |  |
| After 3–24 h | 1.59 (0.72–3.50) | .25 |
| After 24–48 h | 1.12 (0.46–2.76) | .80 |
| Delayed >48 h | 0.94 (0.29–3.07) | .91 |

Note. CI, confidence interval.

**Supplementary Table 3.** Source of Infection Among Patients with Low, Medium, and High Pitt Bacteremia Scores^a^

| Source of Infection | Low  (n=54), No. (%) | Medium  (n=69), No. (%) | High  (n=36), No. (%) |
| --- | --- | --- | --- |
| Central line | 12 (22) | 8 (12) | 3 (8) |
| Urinary system | 4 (7) | 13 (19) | 0 (0) |
| Nonurinary intra-abdominal | 10 (19) | 10 (14) | 8 (19) |
| Pneumonia | 4 (7) | 6 (9) | 12 (33) |
| Multiple sources | 2 (4) | 3 (4) | 1 (3) |
| Other | 9 (17) | 8 (12) | 7 (19) |
| Unknown | 13 (24) | 21 (30) | 6 (17) |

^a^Pitt bacteremia scores of zero were considered low, scores 1–4 were considered medium, and scores >4 were considered high. Overall distribution of source of infection is significantly associated with Pitt category based on the Fisher exact test (*P* = .006).
